# Impact of rheological properties on bacterial streamer formation

**DOI:** 10.1098/rsif.2021.0546

**Published:** 2021-10-20

**Authors:** Hiroki Kitamura, Toshihiro Omori, Takuji Ishikawa

**Affiliations:** ^1^ Department of Finemechanics, Tohoku University, Aoba 6-6-01, Sendai, Miyagi, Japan; ^2^ Department of Biomedical Engineering, Tohoku University, Aoba 6-6-01, Sendai, Miyagi, Japan

**Keywords:** biofilm, streamer, biofluid

## Abstract

Bacterial biofilms, which can be found wherever there is water and a substrate, can cause chronic infections and clogging of industrial flow systems. Despite intensive investigation of the dynamics and rheological properties of biofilms, the impact of their rheological properties on streamer growth remains unknown. We numerically simulated biofilm growth in a pillar-flow and investigated the effects of rheological properties of a filamentous flow-shaped biofilm, called a ‘streamer’, on its formation by varying the viscoelasticity. The flow-field is assumed to be a Stokes flow and is solved by a boundary element method. A Maxwell model is used for extracellular matrix-mediated streamer growth to express the fluidity of streamer formations. Both high elastic modulus and viscosity are needed for streamer formation, and high viscosity promotes streamer growth at low cell concentrations. Our findings are consistent with experimental observations and can explain the relationship between the cell concentrations and viscosity at which streamers form.

## 1. Introduction

Biofilms are colonies of bacteria encased in a self-excreted matrix of extracellular polymeric substances (EPS) [1,2]. In nature, biofilms can be found on rocks, plant surfaces, soil, etc. wherever there is water and a substrate [3,4]. EPS act as a barrier and carrier for the biofilm, protecting the bacteria inside from chemical and physical stimuli [5]. Bacteria within biofilms can communicate to control their growth rate via quorum sensing [6]. Biofilm structures can thus result from the interaction between the environment and individual cells.

Hydrodynamics also influence biofilm formation, because they govern drag force and mass transport [7–10]. A remarkable example of this is filamentous flow-shaped biofilms, called ‘streamers’ [11]. Streamers are frequently observed in soil-like porous environments and industrial filters. The impact of fluid flow on streamer formation has been studied using microfluidic devices [2,11–14]. Drescher *et al.* [12] reported *Pseudomonas aeruginosa* biofilms to form three-dimensional streamers, which over time bridge the gaps between obstacles and corners in non-uniform environments. This bridging has also been reported for *Pseudomonas fluorescens* [13] and *Escherichia coli* streamers [14]. Using two types of fluorescence-expressing cells, Drescher *et al.* [12] also showed that the growth of the streamer was due to the capture of advecting cells, and not by wall-attached cell growth, and concluded that flow-induced shedding of the extracellular matrix from surface-attached biofilms generates a sieve-like network that exponentially accelerates clogging. Rusconi *et al.* [11] showed biofilm streamer formation concentrates in the middle plane of curved microchannels, and that streamer formation is characterized by the intensity of secondary flow around corners [2]. They also made time-lapse observations of streamers in the early stages of development. Initially, the streamers consisted only of EPS and gradually developed into thicker streamers containing bacteria. Taherzadeh *et al.* [15] numerically investigated oscillatory movement of biofilm streamers in high Reynolds number flow. They showed that formation of a Kármán vortex street behind the streamer body is the main source of the periodic oscillation of the streamers. Xia *et al.* [16] also numerically investigated cohesive failure of two oscillating streamers in parallel and tandem arrangements.

A number of studies on the rheological properties of biofilms using several measurement techniques, including rheometry, uniaxial compression, atomic force microscopy and hydrodynamic load, indicated that biofilms are viscoelastic [4,17–21]. The elastic modulus and viscosity of biofilms vary widely between cell types and growth stages, with shear modulus ranging from 10^−2^ to 10^6^ Pa [1]. Shaw *et al.* [4] investigated the relaxation time of *Streptococcus mutans* and *P. aeruginosa* biofilms with the rheometer creep test. The best fit of log–log data clarified the relationship of log *η* = 1.03 log *G* + 3.04, resulting in an effective relaxation time *τ* ∼ 1100 s, where *η* is the effective viscosity and *G* is the effective shear elastic modulus. Stotsky *et al.* [21] developed a computational model of biofilms and investigated the complex shear moduli, *G*′ and *G*″. They concluded that treating the spring-like connections between bacteria as Maxwell or Zener elements provides good agreement with the mechanical properties. Jones *et al.* [22] also succeeded in reproducing the viscoelastic properties of *Staphylococcus epidermidis* biofilms by Burger models. These biomechanical studies have led to a consensus on the use of viscoelastic fluid models to describe the viscoelastic properties of biofilms in early development.

To simulate biofilm formation, several mechanical models have been developed. There are two major types of biofilm models: individual particle-based and continuum multiphase flow models. A particle-based biofilm model is focused on individual cell behaviours in an extracellular matrix [21,23–26]. Particle model is based on a mechanistic theory and is used to predict emergent mechanical response of biofilms [23,24]. In a particle model, cells are typically represented by Lagrangian spheres in a Eulerian liquid domain. These particle-based models reproduced three-dimensional multi-species biofilm formation and predicted the spatio-temporal distributions of bacteria and substrates [25,26]. In continuum multiphase flow modelling [27–29], on the other hand, bacterial biofilms are modelled as a polymer solution immersed in a Newtonian liquid. Biofilm motion and growth are described by mass conservation of the polymer phase, solvent phase and nutrients, as well as by momentum conservation in both the polymer and solvent phases.

Biomechanical modelling of biofilms has revealed streamer growth in microfluidics and the viscoelastic properties of biofilms [4,11,12,14,21,22], but it is unclear how these relate to the formation of biofilms. In this study, we numerically investigate the effects of the rheological properties of biofilm on streamer formation. In the following section, we derive governing equations and numerical methods for bacterial motion. In §3, we show the effects of rheological properties on streamer growth, and provide conclusions in §4.

## 2. Governing equations and numerical methods

### 2.1. Problem setting

**Figure 1.**
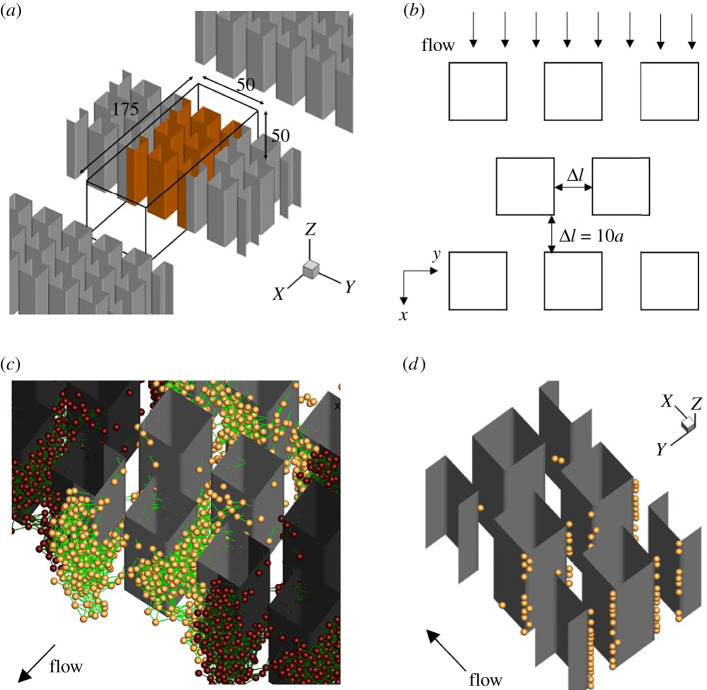
Problem settings. (*a*) Triply periodic boundary is given for a fluid domain of 175*a* × 50*a* × 50*a*. The main fluid domain is highlighted in the figure. (*b*) Each square pillar is set to be 15*a* × 15*a*, and each pillar-to-pillar distance Δ*l* is set to 10*a*. (*c*) Bacterial cells flow from upstream (*x* = 0) under a random distribution with a constant interval, and are excluded from the calculation when they are out from the downstream boundary (*x* = 175*a*). EPS-mediated cell–cell adhesions are modelled by a Maxwell model, which are coloured by green. (*d*) Distributions of 100 pre-defined wall-adherent cells.

To investigate streamer formation in a microfluidic system, we set up the channel geometry as shown in figure 1*a*. The triply periodic boundary conditions are given for a fluid domain of 175*a* × 50*a* × 50*a*, as highlighted in the figure, where *a* is the radius of a bacterium (typically 1 μm). To mimic the experiment of Marty *et al.* [14], the size of each square pillar is set to 15*a* × 15*a* and each pillar-to-pillar distance Δ*l* is set to 10*a* (cf. figure 1*b*). The uniform background velocity of *U* in the *x*-direction is given as the boundary condition on top of the triply periodic boundary conditions.

Bacterial cells flow from upstream (*x* = 0) under a random distribution with a constant interval, and are excluded from the calculation when they are out of the downstream boundary (*x* = 175*a*). We neglect swimming of bacteria throughout this study, i.e. the cells are modelled as passive flowing particles, because the typical average velocity *U* is sufficiently large compared to the bacterial swimming speed *U*_*b*_ (e.g. *U* ∼ 4.5 mm s^−1^ [14], *U* ∼ 1 mm s^−1^ [2,11], *U*_*b*_ ∼ 10 μm s^−1^ [30]). Drescher *et al.* [12] reported that the main factor in streamer growth is cell/extracellular matrix trapped in the mesh structure of EPS, independent of cell growth. We ignore cell division during the computation and assume streamers grow via the adhesion of flowing cells.

Bacteria adhere to the walls of the vessel, or to other bacteria via EPS, when they are close to each other; this adhesion is broken when they are separated from each other. EPS-mediated cell–cell adhesions are modelled by a Maxwell model (cf. figure 1*c*). The cell–wall adhesion is not taken into account during the calculation, excluding pre-defined wall-adherent cells. Simulations are performed with 100 pre-defined wall-adherent cells (cf. figure 1*d*) as the initial conditions for streamer growth. More information on how to set wall-adherent cells and measure the impact of initial conditions is provided in appendix A.

### 2.2. Fluid mechanics

Next, we describe the governing equations of fluid motion. The working fluid is assumed to be an incompressible Newtonian fluid with density *ρ* and viscosity μ. The Reynolds number around the cell is much smaller than unity (*Re* = *ρU a*/μ ∼ 10^−3^), and we assume that fluid motion is governed by the Stokes equation. Far-field interactions between cells are taken into account, and fluid flow at any point x can then be given by the boundary integral equation [31–33]:

2.1 $$v(x)=v^{\infty }+\frac{1}{8\pi \mu }\sum_{\lambda =0}^{\infty }\int_{\mathrm{wall}}J^{E}(x,y_{\lambda })\cdot q^{w}(y_{\lambda })dS(y_{\lambda })+\frac{1}{8\pi \mu }\sum_{\lambda =0}^{\infty }\sum_{i}^{N}J^{E}(x,X_{\lambda }^{i})\cdot F_{\lambda }^{i},$$

where $v^{\infty }=(U,0,0)$ is the background flow, $q^{w}$ is the traction acting on the pillar surface and $X^{i}$ and $F^{i}$ are the material point and force acting on the *i*th cell, respectively. The subscript *λ* indicates the periodic lattice and point $y_{\lambda }$ is equivalent to $y_{\lambda }=y+\lambda b$ (same for $X^{i}$ and $F^{i}$), where b is the lattice vector defined as $b=ib_{x}+jb_{y}+kb_{z}$ (three integers *i*, *j*, *k* take the values −1, 0 or 1, except for the special case *i*, *j*, *k* = 0, 0, 0). Three base vectors $b_{i}$ are given by $b_{i}=L_{i}e_{i}$ (without taking Einstein’s summation), where *L*_*i*_ and $e_{i}$ are the domain size and Cartesian basis vector in the *i*-direction, respectively.

To obtain fast convergence of equation (2.1), we employ an Ewald summation. According to Beenakker’s work [32], Green’s function $J^{E}$ is decomposed into two terms using the error functions: $J^{E}=J^{I}+J^{II}$, where

2.2 $$\begin{array}{r} \left[\begin{array}{c} J^{I}\\ J^{II} \end{array}\right]=(I\nabla^{2}-\nabla \nabla )\left[\begin{array}{c} r\;\mathrm{erfc}(\xi r)\\ r\;\mathrm{erf}(\xi r) \end{array}\right], \end{array}$$

*r* is the distance between the source point $y_{\lambda }$ and observation point x, $r=|x-y_{\lambda }|$, I is the identity matrix and *ξ* is an arbitrary positive constant with dimensions of inverse length. For a simple cubic lattice, the optimal value of *ξ* can be given by $\sqrt{\pi }{/}^{3}\sqrt{V}$ [32], where *V* is the domain volume. We then set $\xi =\sqrt{\pi }{/}^{3}\sqrt{V}$ throughout this study. After straightforward algebra, we obtain

2.3 $$J^{I}=E_{1}\frac{I}{r}+E_{2}\frac{rr}{r^{3}}$$

and

2.4 $$J^{II}=\frac{8\pi E_{3}}{V}\left(\frac{I}{k_{\lambda }^{2}}-\frac{k_{\lambda }k_{\lambda }}{k_{\lambda }^{4}}\right)\;\mathrm{when}\;\lambda \neq 0,$$

where

2.5 $$E_{1}=\mathrm{erfc}(\xi r)+\frac{2\xi r}{\sqrt{\pi }}(2\xi^{2}r^{2}-3)\exp(-\xi^{2}r^{2}),$$

2.6 $$E_{2}=\mathrm{erfc}(\xi r)+\frac{2\xi r}{\sqrt{\pi }}(1-2\xi^{2}r^{2})\exp(-\xi^{2}r^{2})$$

2.7 $$\mathrm{and}\;E_{3}=\left(1+\frac{k_{\lambda }^{2}}{4\xi^{2}}+\frac{k_{\lambda }^{4}}{8\xi^{4}}\right)\exp\left(-\frac{k_{\lambda }^{2}}{4\xi^{2}}\right)\exp\left(ik_{\lambda }\cdot \hat{r}\right),$$

where $\hat{r}=x-y_{\lambda =0}$ and $k_{\lambda }$ is the reciprocal lattice vector defined as $k_{\lambda }\cdot b=2\pi \lambda$.

### 2.3. Bacterial adhesion and dissociation

We next explain the model for bacterial adhesion and detachment. Bacterial adhesions between two cells are assumed to be viscoelastic adhesions with EPS bridges, which occur immediately when they are close to each other. Previous studies on the biomechanics of biofilms have investigated their viscoelastic properties [21,22]. These studies have led to the conclusion that models representing viscoelastic fluid properties, such as Maxwell, Burger and Zener models, are suitable for representing rheological properties in the early stage of biofilms. Viscoelastic bacterial adhesion is then modelled as a Maxwell model and represented by springs and dampers. In the Maxwell model, total displacement is expressed as the sum of spring and damper displacements, and the adhesive force is equal to the spring and damping forces

2.8 $$\Delta X=\Delta X_{1}+\Delta X_{2}$$

and

2.9 $${\;f}_{ij}^{ad}=k\Delta X_{1}=c\frac{dX_{2}}{dt},$$

where Δ*X* is the total displacement of the viscoelastic element, ${\;f}_{ij}^{\mathrm{ad}}$ is the adhesive force between cells *i* and *j*, *k* is the spring constant, *c* is the damping coefficient and Δ*X*_1_ and Δ*X*_2_ are spring and damper displacements, respectively. The natural length of the spring is the intercellular distance at the start of adhesion, and the initial damper length is zero. Total adhesive force acting on the *i*th cell can be given by

2.10 $$F^{i}=\sum_{\;j}^{n}{\;f}_{ij}^{\mathrm{ad}},$$

where *n* is the number of adherent cells. New adhesion is determined by the distance within the threshold *r*_ad−cell_ and the threshold used for desorption is *r*_det−cell_. These values are treated as parameters, the results of which are given in the Results section.

### 2.4. Numerical procedure

To simulate streamer formation in the microchannel, the pillar surface is discretized by *N*_*e*_ triangles with *N*_*n*_ vertices, where *N*_*e*_ is the number of elements and *N*_*n*_ is the number of nodes. Due to the no-slip condition, the velocity v is zero at the pillar surface; v(x)=0 with $x\in S_{\mathrm{wall}}$. Equation (2.1) is then rewritten as the following vector form:

2.11 $$\left[\begin{array}{c} J \end{array}\right]\left[\begin{array}{c} q^{w} \end{array}\right]=\left[\begin{array}{c} -v^{\infty }-v^{\mathrm{cell}} \end{array}\right]\;(x\in S_{\mathrm{wall}}).$$

The matrix J is numerically calculated with finite *λ* using a Gaussian numerical integration scheme [34]. The matrix size is 3*N*_*n*_ × 3*N*_*n*_, and we preliminarily calculated the inverse matrix of J with an LU factorization technique [35]. We note that J in equation (2.11) is invariant with time, as both observation points and source points are defined at the wall surface. Once $J^{-1}$ is given, we calculate $q^{w}$ at every time-step by multiplying it by the right-hand side of equation (2.11). $v^{\mathrm{cell}}$ is the flow generated by the cellular adhesive force, which is as time-variant as streamer growth. The velocity of *i*th cell is also calculated according to the following equation of motion:

2.12 $$\begin{array}{rl} \frac{dX^{i}}{dt}=v^{i}(X^{i}) & =v^{\infty }+\frac{1}{8\pi \mu }\sum_{\lambda =0}^{\lambda_{\max}}\int_{\mathrm{wall}}J^{E}\cdot q^{w}dS\\ & \;+\frac{1}{6\pi \mu a}F^{i}+\frac{1}{8\pi \mu }\sum_{\lambda =0}^{\lambda_{\max}}\sum_{\begin{array}{c} j\neq i\\ \mathrm{when}\;\lambda =0 \end{array}}^{N}J^{E}\cdot F_{\lambda }^{j}. \end{array}$$

After the velocity is given, the cellular position $X^{i}$ is updated by a second-order Runge–Kutta method, as well as adhesive forces according to equations (2.9) and (2.10). All procedures are repeated until we reach the desired computational time.

We preliminarily checked the convergence $q^{w}$ by changing *λ*_max_ without $v^{\mathrm{cell}}$, and confirmed that the result does not change as much when *λ*_max_ = 3 or higher (less than 1%). We also checked the mesh convergence and numerically confirmed that *N*_*e*_ = 11 520 and *N*_*n*_ = 6132 (*N*_*e*_ = 1920 and *N*_*n*_ = 1022 for each pillar) are sufficiently high to resolve the flow in the microchannel. We then decided to use *λ*_max_ = 3, *N*_*e*_ = 11 520 and *N*_*n*_ = 6132 in this study.

All parameters are non-dimensionalized by the background velocity *U*, viscosity μ and cellular radius *a*: e.g. time t* = *tU*/*a*, force f* = *f*/*μaU*. Hereafter, non-dimensionalized physical quantities are denoted by asterisks. Units and ranges for each parameter are shown in table 1.

**Table 1.** Units and ranges for each parameter in the SI system of units. All parameters are non-dimensionalized by the inlet velocity *U*, fluid viscosity μ and the cell radius *a*.

parameter	unit	dimensionless	parameter range
spring constant *k*	N m^−1^	k* = *k*/*μU*	k* ∈ [10, 100]
damping coefficient *c*	N s m^−1^	c* = *c*/*μa*	c* ∈ [10, 10 000]
bonding radius *r*_ad−cell_	m	$r_{\mathrm{ad}-\mathrm{cell}}^{*}=r_{\mathrm{ad}-\mathrm{cell}}/a$	$r_{\mathrm{ad}-\mathrm{cell}}^{*}\in [2.75,4.0]$
detachment radius *r*_det−cell_	m	$r_{\det-\mathrm{cell}}^{*}=r_{\det-\mathrm{cell}}/a$	$r_{\det-\mathrm{cell}}^{*}\in [6.0,18.0]$
time *t*	s	t* = *tU*/*a*	
force *F*	N	F* = *F*/*μaU*	
traction on the wall *q*^*w*^	Pa	*q*^w*^ = *q*^*w*^*a*/*μU*	
streamer length *l*_st_	m	$l_{\mathrm{st}}^{*}=l_{\mathrm{st}}/a$	
Young’s modulus of a streamer *E*	Pa	E* = *Ea*/*μU*	
viscosity of a streamer *μ*_*s*_	Pa s	$\mu_{s}^{*}=\mu_{s}/\mu$	

## 3. Results and discussion

### 3.1. Streamer formations

**Figure 2.**
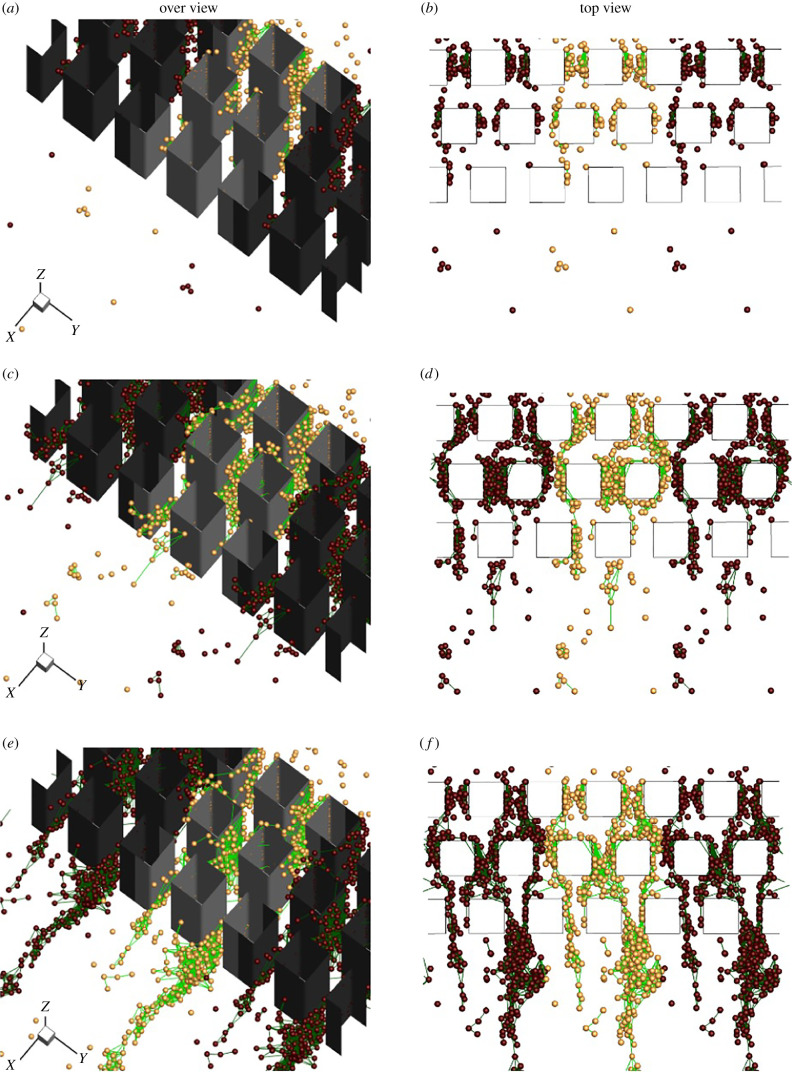
Streamer formation in a microchannel. The left-hand panels are overviews and the right-hand ones are top views: (*a*,*b*) t* = 120, (*c*,*d*) t* = 240 and (*e*,*f*) t* = 400. Note that periodic images are depicted by shade colours.

We first investigate the time evolution of streamer formation under pillar flow. The spring constant k* (=*k*/*μU*) and damping coefficient c* ( = *c*/*μa*) are set to k* = 100.0 and c* = 1000.0, respectively. The attachment and detachment threshold radii are set to $r_{\mathrm{ad}-\mathrm{cell}}^{*}=3.0$ and $r_{\det-\mathrm{cell}}^{*}=12.0$. Simulations are performed during t* ∈ [0, 1600] and the results are shown in figure 2 and electronic supplementary material, movie S1.

After starting the calculation, bacteria arrive from upstream with a random distribution at a constant influx rate, i.e. the number of influx cells per unit time per unit cross-sectional area is constant at 4 cells per unit time per unit area. In this simulation, we assume that cells that adhere tightly to the wall generate EPS, and model the adhesion force as a viscoelastic force expressed by the Maxwell model. Adhesion of the viscoelastic body is assumed to occur by the uptake of EPS by cells within the threshold distance from the cells attached to the wall. When flowing cells approach wall-adherent cells closer than $r_{\mathrm{ad}-\mathrm{cell}}^{*}$, viscoelastic adhesion occurs between the cells. Once bonded to wall-adherent cells, the cells are assumed to develop the ability to produce EPS, which allows the bonding cells to adhere to other flowing cells. Streamers then begin to grow in parts of the channel, as shown in figure 2*a*,*b*. The growing streamer flows in a downstream direction and forms a bridge from upstream and downstream pillar (cf. figure 2*d*,*f*). The bridging of streamers from wall corner to wall corner has been observed in previous experiments [11,12,14] and is in qualitative agreement with the results. We tested another boundary condition that allows cells to adhere to the wall. In this case, the streamer grew upstream to cover the entire channel, and the bridging observed in the experiments did not occur (see appendix A). The streamer continued to grow downstream of the third row, forming an elongated streamer in the downstream region (cf. figure 2*e*,*f*). Once the streamer had grown sufficiently, dissociation from the wall occurred and it was swept downstream (see electronic supplementary material, movie S1). Such dissociation has been observed experimentally [14], and repeated growth and dissociation were observed in the simulation. Though repeated break-off events were observed in scummy biofilm streamers, majority of them do not. Many bacteria species can form very long streamers containing very few cells. For example, *Pseudomonas* sp. can rapidly form onset streamer filament at an oil–water interface with only one or two cells attached to a very thin EPS filament (about 200 nm in diameter but several millimetres in length) [36]. When modelling long EPS filaments, interactions between viscoelastic deformation of filaments and fluid flow need to be taken into account. The current model would therefore be limited to the growth of scummy biofilms with densely packed cells (short filament distances).

**Figure 3.**
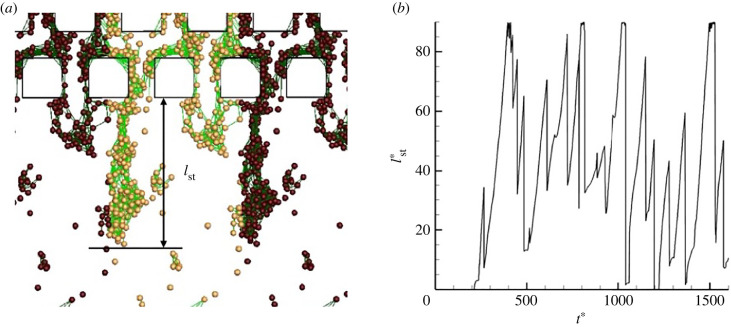
Streamer length. (*a*) Definition of the streamer length *l*_st_ downstream of the third row and (*b*) time change of *l*_st_.

To measure streamer growth quantitatively, we measure the streamer length *l*_st_ downstream of the third row, as shown in figure 3. The time change of *l*_st_ is shown in the figure, and it can be seen that streamers tend to grow monotonically in the early stage of their formation (t* ≤ 400). The streamer reaches the downstream boundary ${\left(l_{\mathrm{st}}^{\max}\right)}^{*}=90$ and no further growth can occur, but after the streamer has grown sufficiently, it dissociates from the wall and the *l*_st_ decreases rapidly. Repeated growth and dissociation are observed throughout the simulation, as shown in figure 3*b*. Marty *et al.* [14] reported that the average length of *E. coli* streamers in pillar flow is about 10–100 μm. Our numerical results are also of the order of 10 μm, in quantitative agreement with the experimental results of Marty *et al.* [14]

### 3.2. Effect of critical distance of cellular adhesion/detachment

We next investigate the effects of the adhesion and detachment threshold *r*_ad−cell_ and *r*_det−cell_. In contrast to firm adhesion to the substrate surface, EPS-mediated cell–cell adhesion is highly fluidic in streamer formation. The average intercellular distance of *Klebsiella pneumoniae* biofilms formed in a static fluid environment is a few micrometres [37], and Stotsky *et al.* [21] determined *r*_ad−cell_ to be 1.62 μm. For the detachment *r*_det−cell_, values of the same order as the adhesion distance were used in previous numerical studies [21]. However, the critical values can be varied according to the cell type and growth stage. Then, *r*_ad−cell_ and *r*_det−cell_ are treated as parameters, and these are changed as $r_{\mathrm{ad}-\mathrm{cell}}^{*}\in [2.75,4.0]$ and $r_{\det-\mathrm{cell}}^{*}\in [6.0,18.0]$. The distance *r* is calculated as the distance from centre to centre, and a distance r* = 2.0 indicates that two cells are in contact.

**Figure 4.**
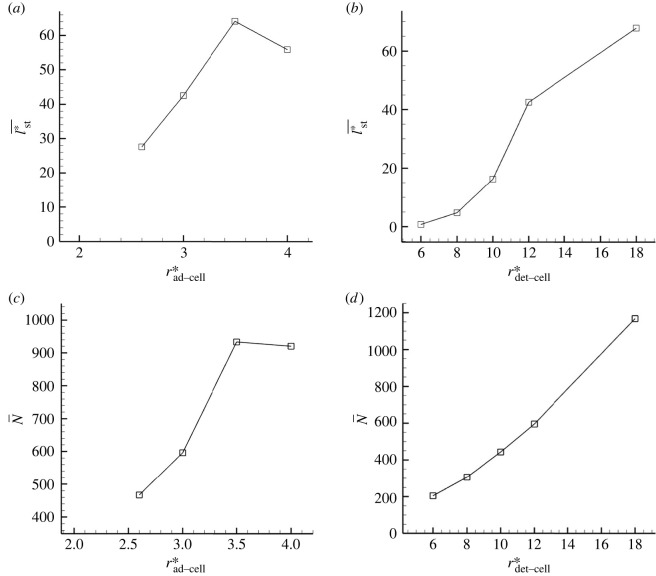
Effect of adhesion and detachment threshold *r*_ad−cell_ and *r*_det−cell_. (*a*,*c*) Time averaged streamer length *l*_st_ and the time averaged number of adherent cells *N* as a function of *r*_ad−cell_, whereas *r*_det−cell_ in (*b*,*d*).

Time-averaged streamer length ${\bar{l}}_{\mathrm{st}}^{*}$ with various $r_{\mathrm{ad}-\mathrm{cell}}^{*}$ is shown in figure 4*a*. In this case, the critical detachment distance is fixed to $r_{\det-\mathrm{cell}}^{*}=12.0$. Streamers tend to grow thicker and longer with increasing $r_{\mathrm{ad}-\mathrm{cell}}^{*}$. The probability of cell adhesion increases with adhesion distance $r_{\mathrm{ad}-\mathrm{cell}}^{*}$, so large numbers of adherent cells are observed with increasing $r_{\mathrm{ad}-\mathrm{cell}}^{*}$ (cf. figure 4*c*). When the attachment distance is sufficiently large, $r_{\mathrm{ad}-\mathrm{cell}}^{*}>3.5$, a large population of cells is rapidly formed in the upstream region; they break off before they can grow in the downstream region, causing the streamer length to reach a plateau. On the other hand, when $r_{\det-\mathrm{cell}}^{*}$ increases, both average streamer length ${\bar{l}}_{\mathrm{st}}^{*}$ and number of adherent cells $\bar{N}$ increase monotonically, as shown in figure 4*b*,*d*. As the detachment distance increases, floating cells tend to attach one after the other before detaching, resulting in the formation of a long streamer. If we set $r_{\det-\mathrm{cell}}^{*}=\infty$, the streamer should not dissociate and continue to grow, but this tendency is different from the experimentally observed repeated growth and dissociation of the streamer [14]. The growth rate of the streamer, which depends on the detachment distance, is strongly dependent on the frequency of cell influx and viscosity of the streamer. The relationships among them are discussed in §3.4.

### 3.3. Effects of spring constant and damping coefficient

**Figure 5.**
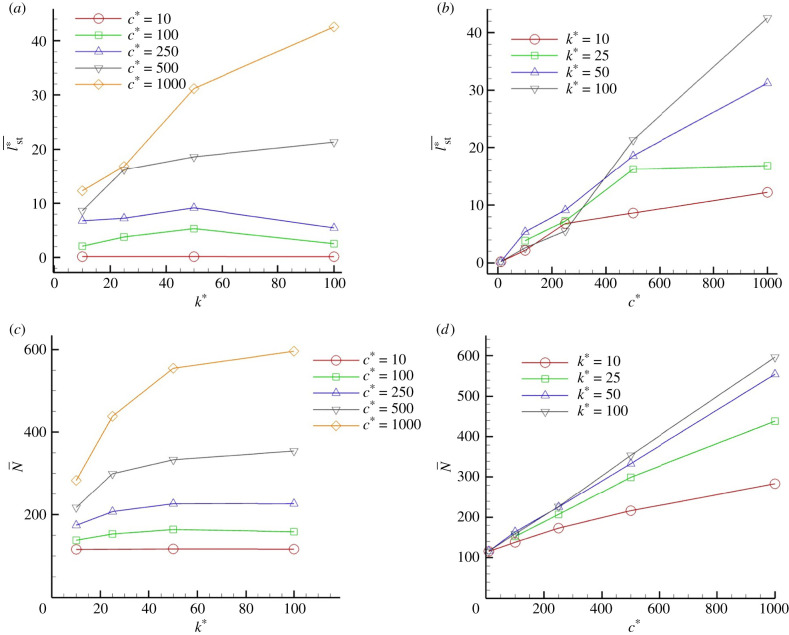
Effect of spring constant and damping coefficient *k* and *c*. (*a*,*c*) Time averaged streamer length *l*_st_ and the time averaged number of adherent cells *N* as a function of *k*, whereas *c* in (*b*,*d*).

The effects of the spring constant and damping coefficient are shown in figure 5. With a weak damper (cf. c* ≤ 500 in figure 5*a*,*c*), time-averaged adherent cells, and the average length of the streamer, are almost invariant with the spring constant. On the other hand, with a strong damper (c* = 1000), the streamer length increases monotonically with the spring constant. With a strong damper, the viscous effect becomes dominant, which lengthens the time required to reach the detachment distance. Accordingly, new adhesion of the flowing cells should promote growth of the streamer.

When the spring constant is fixed and the damper coefficient is varied, both the streamer length and number of adherent cells are increased monotonically with the damping coefficient, as shown in figure 5*b*,*d*. It can also be seen that the length of the streamer increases with the spring constant and damping coefficient. These results indicate that both a large spring constant and large damping coefficient are necessary for the formation of streamers, and it is assumed that streamers cannot be formed by either. This may be because a low spring constant reduces the adhesive force, so that the bacteria cannot resist the shear force when they clump together. Moreover, a low damping factor causes the adhesion to stretch quickly, so that the bacteria are released faster than they can be added. This implies that the viscoelastic properties of the streamer govern its growth. It is necessary to investigate the macroscopic rheological properties of the streamer, such as the relaxation time, Young’s modulus and viscosity. These effects are discussed in the next section.

### 3.4. Effects of the rheological properties of a streamer on its growth

To discuss the effects of the rheological properties of a streamer on its growth, it is necessary to develop a method to calculate the macroscopic properties from behaviours at the cellular scale. As it is difficult to derive a three-dimensional constitutive law for streamers, in this study, we focus only on deformations and forces in the mainstream direction, and assume that the streamer is elongated in one dimension. We then calculate Young’s modulus *E* and elongational viscosity μ_*s*_ from the simulation. Details of the methodology are presented in appendix A.

The results are shown in figure 7. Young’s modulus of a streamer is almost proportional to the spring constant of the cell–cell adhesion force, and the damping coefficient of the cell–cell adhesion force has no significant influence on its value. The estimated non-dimensional Young’s modulus of a streamer is about E* (= *Ea*/*μU*) ∈ [6, 54]. These values are equivalent to *E* ∼ 25 to 243 Pa, assuming that the cell radius *a* = 1 μm, fluid viscosity μ = 1 mPa s and characteristic fluid velocity *U* = 4.5 mm s^−1^ [14]. Although Young’s modulus of biofilms varies widely, the values of *P. aeruginosa* streamers and early *Bacillus subtilis* biofilms are reported to be around 70–140 Pa [2] and 300 Pa [19], respectively. We then confirmed quantitative agreement with these previous experiments.

The elongational viscosity of a streamer is also proportional to the damping coefficient of cell–cell adhesion force and the spring constant has no significant influence on its value. The non-dimensional viscosity $\mu_{s}^{*}\;(=\mu_{s}/\mu )$ is up to 600, which can be estimated as *μ*_*s*_ is up to 0.6 Pa s. This value is much smaller than the typical viscosity of bacterial biofilms: 10^3^ to 10^5^ Pa s [2,4,18,20]. Accordingly, the relaxation time in the present study is much smaller than the experimental results: 0.01 s versus 10^3^ s, respectively. That is, the timescale for streamer growth in the present study is of the order of a few seconds, whereas that in the experiment is about a day. We tried to simulate streamer formation with high damping coefficient, but the calculations became unstable due to the high degree of cell aggregation. With high damping coefficient, cell migration speed during the aggregation decayed quickly. This resulted in a large number of cells staying in the channel, forming large clusters and making the calculation unstable. To avoid the instability, the influx cell rate should be decreased according to the damping coefficient, but this leads to slow streamer development and takes 10 to 100 times longer computation time, making it difficult to obtain full simulation results. As it is difficult to perform calculations on a 24 h scale with millisecond resolution, we discuss the effects of viscosity by extrapolating the results of cell influx frequency and damping coefficient.

As described in §3.3, the streamer growth rate is affected by both the cell influx frequency and damping coefficient. With a weak damper and low influx frequency, streamers elongate quickly, so that the streamers flow downstream faster than bacteria can be added. By contrast, under conditions where both the damper and feed rate are high, it is likely that the streamer would not grow downstream but instead clog the upstream flow path. It can therefore be inferred that there is an effect of the relationship between cell influx and viscosity on streamer growth.

**Figure 6.**
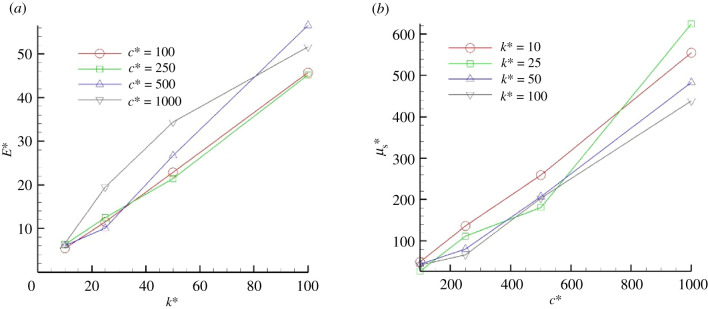
Viscoelastic properties. (*a*) Young’s modulus of a streamer as a function of spring constant of cell–cell adhesion force, and (*b*) extension viscosity of a streamer as a function of damping coefficient of cell–cell adhesion force.

**Figure 7.**
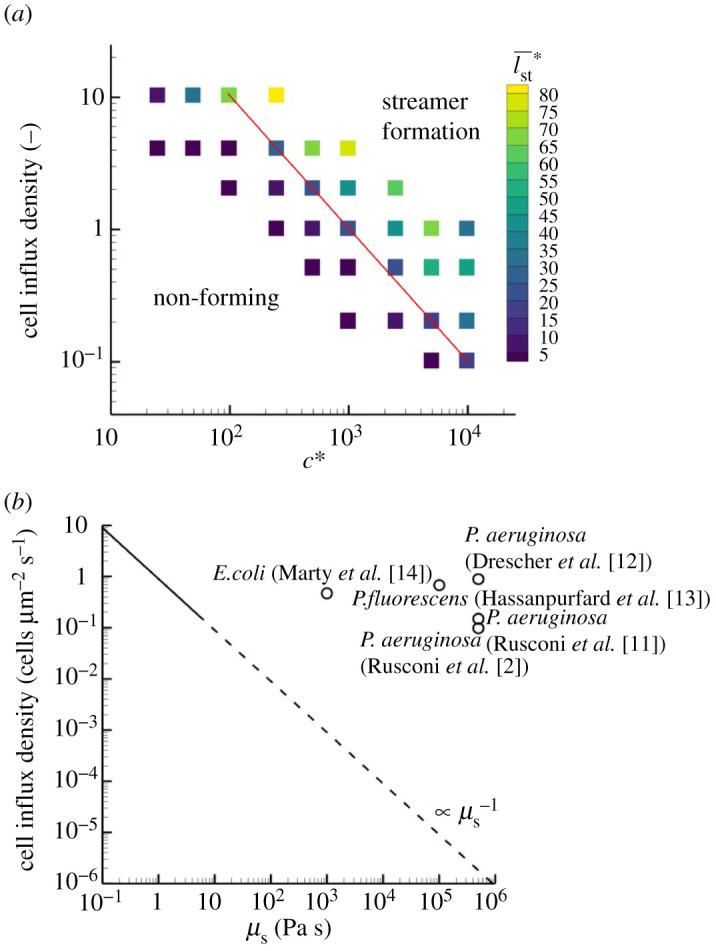
Streamer growth in influx cell concentration and viscosity space. (*a*) Phase diagram in cell concentration and damping coefficient space. The red line shows the threshold of ${\bar{l}}_{\mathrm{st}}^{*}\geq 20$. (*b*) Comparison of present results with experimental conditions by extrapolation. Each plot is given from [2,11–14].

The relationships between the cell influx density (number of cells per unit cross-sectional area per unit time) with various damping coefficients are shown in figure 7*a*. Streamers can be formed only in the strong damper and high influx regime region. The average length of the streamer is used as a criterion for determining whether a streamer will grow or not; the area where the average length is 20*a* is shown in the figure. It can be seen that the threshold boundary is proportional to *c*^−1^ (red line in the figure); based also on the previous results (cf. figure 6*b*), it is inversely proportional to the viscosity. Assuming that this linear relationship is valid in the high-viscosity regime, the extrapolated result is shown in figure 7*b*. For comparison, the experimental conditions of *P. aeruginosa* [2,11,12], *P. fluorescens* [13,20] and *E. coli* [14,18] are also plotted in the figure. All experimental data are plotted above the threshold, and the similarity can be seen between the present results and the experimental ones. These results indicate that high viscosity or long relaxation times of streamers, i.e. the viscous-dominant rheological properties of early biofilms, aid streamer growth at low cell concentrations and promote the initial growth of biofilms.

## 4. Conclusion

In this study, we developed a computational model of streamer formation in a microchannel. Macroscopic rheological properties are reconstructed from cell-scale adhesion phenomena, and we discuss their impact on streamer growth. We found both high elastic modulus and viscosity are needed for streamer formation; in particular, high viscosity aids streamer growth at low cell concentrations. These findings improve our understanding of streamer growth in microfluidics, industrial filters and some medical devices. It has been reported that streamers exhibit a nonlinear viscoelastic response to large deformations [38], suggesting that nonlinear behaviour can affect the fracture of streamer growth [17]. Further analysis of the nonlinear response of streamers should be required in the future.

## Figures and Tables

**Figure 8.**
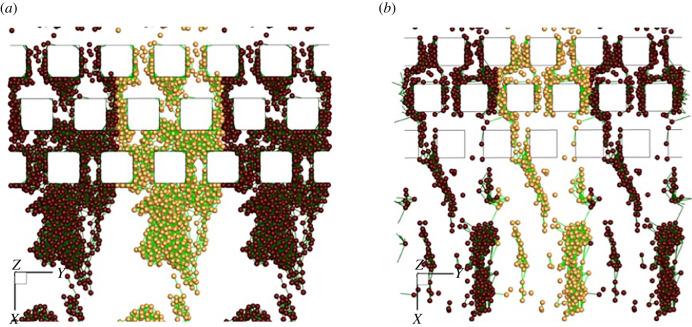
Effect of wall–cell adhesion: In (*a*), all bacteria can adhere to the wall, whereas in (*b*), the cells that attach to the wall are predefined and planktonic cells cannot attach to the wall.

**Figure 9.**
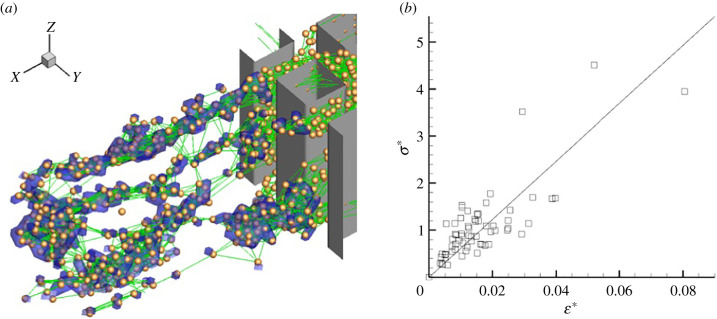
Stress–strain relationship of the streamer. (*a*) Eulerian streamer grid, depicted as blue colour, is defined as a grid where bacteria are connected to the wall surface. (*b*) Young’s modulus is estimated by a least square line approximation in stress–strain relationship. Each plot indicates space average of the numerical result at each time.

## Data accessibility

This article has no additional data.

## Appendix A

### A.1. Effects of initial conditions: pre-defined wall-adherent cells

In this study, we preliminarily defined wall-adherent cells; wall–cell adhesions are not taken into account in the main calculation. Drescher *et al.* [12] reported that biofilm streamers consisted only of flowing cells; biofilm adhering to the wall was not incorporated into the streamer. These results indicate that there is a time delay between the adhesion of wall-adherent and flowing cells. Here, we investigated the effects of wall adhesion on flowing cells, with and without wall adhesion.

To determine wall-adherent cells, we used the same computational domain and boundaries shown in figure 1. We set the wall–cell adhesion radius *r*_ad−wall_ = 1.0*a* and performed computations until the wall-adherent cells reached 100 in the computational domain. The cell influx condition is the same as in figure 2. Most of the bacteria adhere to both corners of the upstream face of the first and second rows of pillars, while only a few adhere to the third row. This is highly consistent with the position where the upstream streamline first approaches the wall.

Figure 8*a* shows that all bacteria can adhere to the wall, and to other cells if they approach within a certain threshold. In this case, the cell population grows to occupy the entire channel, and the streamer grows from the central axis of the pillar. In Figure 8*b*, on the other hand, flowing cells can only attach to other cells, as in figure 2. We can see that the streamer bridges the pillars from corner to corner, and grows along with the streamline. This reproduces the results of previous experiments [11,12,14]; in this study, we assume that wall–cell adhesion occurs only in the cells defined in the preliminary calculations.

Depending on the cell type, biofilm growth is initiated by irreversible adhesion to a substrate, followed by production of EPS, and the viscoelastic properties of the resulting matrix determine its structural integrity, resistance to stress and ease of dispersion [20]. Different experiments on wall–cell adhesion and cell–cell adhesion of *Pseudomonas aeruginosa* have been carried out respectively. Experiments using microbead force spectroscopy have shown that the substrate-cell adhesion pressure is about 20 Pa [20], and experiments using microfluidic channels have shown that the streamer deforms with a viscous stress of about 1 Pa [11]. These results indicate that wall–cell adhesion is several tens of times stronger than cell–cell adhesion. We then assume irreversible adhesion to a substrate for the wall–cell adhesion and pre-defined wall-adhesive cells are fixed during the calculation.

### A.2. Micro–macro relationships: Young’s modulus and elongational viscosity of a streamer

To estimate macroscopic rheological properties, we calculate Young’s modulus and elongational viscosity of a streamer from the numerical simulation. In the downstream region of the channel, the computational domain is divided by a cubic grid of size d*x* × d*y* × d*z*. A grid is defined as a streamer grid if there are bacteria attached to it that are connected to the wall surface (cf. figure 9). As the streamer grid should be about the size of a bacterium, d*x* = d*y* = d*z* = 2.5*a* is chosen in this study.

We compute the tensile stress acting on the streamer grid by summing the adhesive forces of the bacteria within the streamer grid

$$\sigma =\sum_{i}^{n}F_{x}^{i}/A,\;(A\;1)$$

where $F_{x}^{i}$ is the *x*-component of adhesive force acting on the *i*th cell, *n* is the number of adherent cells in the grid and *A* (=d*y* × d*z*) is the cross-sectional area of the streamer grid. In a similar manner, the principal strain is calculated from the average streamer elongation within the grid

$$\varepsilon =\frac{1}{n}\sum_{i}^{n}\varepsilon_{\mathrm{cell}}^{i},\;(A\;2)$$

where *n* is the number of cells within the grid, $\varepsilon_{\mathrm{cell}}^{i}\;(=\sum_{\;j}^{n_{\mathrm{ad}}}\varepsilon^{\;j}/n_{\mathrm{ad}})$ is the average strain of spring elements acting on the *i*th cell and *n*_ad_ is the number of adhesions on the *i*th cell. ɛ^*j*^ is the principal strain of *j*th spring, which is defined as $\varepsilon^{\;j}=((|r^{\;j}|-r^{0})/r^{0}){\hat{r}}^{\;j}\cdot e_{x}$, where $\left|r^{\;j}\right|$ is the length of *j*th spring, *r*^0^ is the natural length and $\hat{r}=r^{\;j}/|r^{\;j}|$. We then calculate the stress and strain averages over the entire downstream region at each time step. The data are plotted on the same graph, as in figure 9, and Young’s modulus is defined from the slope of the least-squares linear approximation at the plotted points. We used the same methodology as for the elongational viscosity.
